# Primary Cilia Orchestrate Cardiac Pathogenesis: A Central Nexus of Remodeling, Signaling, and Repair

**DOI:** 10.1111/cpr.70113

**Published:** 2025-08-25

**Authors:** Yang Yang, Kaidi Ren, Xingjuan Shi, Yi Luan

**Affiliations:** ^1^ Translational Medical Center, Clinical Systems Biology Laboratories The First Affiliated Hospital of Zhengzhou University Zhengzhou P. R. China; ^2^ Department of Pharmacy The First Affiliated Hospital of Zhengzhou University Zhengzhou P. R. China; ^3^ School of Life Science and Technology, Key Laboratory of Developmental Genes and Human Disease Southeast University Nanjing P. R. China

## Abstract

Roles of primary cilia and the signals they transmit in the development of myocardial fibrogenesis, cardiac hypertrophy, and atrial fibrillation. Left, Fibroblasts can differentiate into myofibroblasts in response to TGF‐β1. TGF‐β1 stimulation via both paracrine action in the heart and exogenous action on primary cultured fibroblasts activated the phosphorylation of SMAD3 and the transcription of the fibronectin and collagen type I and III genes. Middle, Vesicles derived from cilia are secreted at an accelerated rate under fluid shear stress. Blockage of ciliary protein, which is required for cELV generation with shRNA, led to blunted cELV secretion and left ventricular hypertrophy. Right, under pathological conditions such as atrial fibrillation (AF), fibroblasts exhibit increased proliferation and differentiation into α‐smooth muscle Actin (αSMA)‐expressing myofibroblasts. This disrupts ECM dynamics, ultimately leading to interstitial fibrosis within the atria. AF patients presented increased HDAC6 activity and reduced levels of acetylated α‐tubulin in left atrial tissues. HDAC6 activity is activated by the interaction of aurora kinase A (AURKA), and neural precursor cells express developmentally downregulated protein 9 (NEDD9) via phosphorylation. LiCl prompts the reversion of αSMA‐positive myofibroblasts into αSMA‐negative fibroblasts.

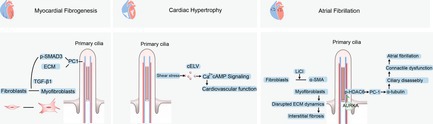

To the Editor,

Cilia are conserved sensory antennae‐like organelles that are microtubule‐based and several micrometres in length. Cilia can receive external stimuli, such as light, chemicals, and mechanical forces, and transfer these signals to cells to initiate corresponding signalling pathways. Cilia can also transmit signals to recipient cells by secreting ectosomes. The functions of cilia are involved in organogenesis, embryonic patterning, and adult tissue homeostasis [1]. Cilia can be classified into two main types based on their movement capabilities and internal structure: motile cilia and nonmotile (primary) cilia. Motile cilia are commonly found in various tissues, including the airway lining, brain ventricles, oviducts, and embryonic nodes. These cilia have an axoneme structure that typically consists of nine pairs of microtubules surrounding a central pair, along with dynein arms and radial spokes. Nonmotile cilia, on the other hand, have a 9 + 0 microtubule arrangement, lacking the central pair, dynein arms, and radial spokes. In most vertebrate cells, a single primary cilium extends from the cell surface. It was previously thought to be a nonfunctional evolutionary remnant. However, recent research has uncovered its vital roles as a mechanosensor and a signalling centre, integrating various signals and playing a key role in tissue development. Dysfunction of both motile and nonmotile cilia is involved in diverse types of human diseases, known as ciliopathies (Figure 1).

**FIGURE 1:**
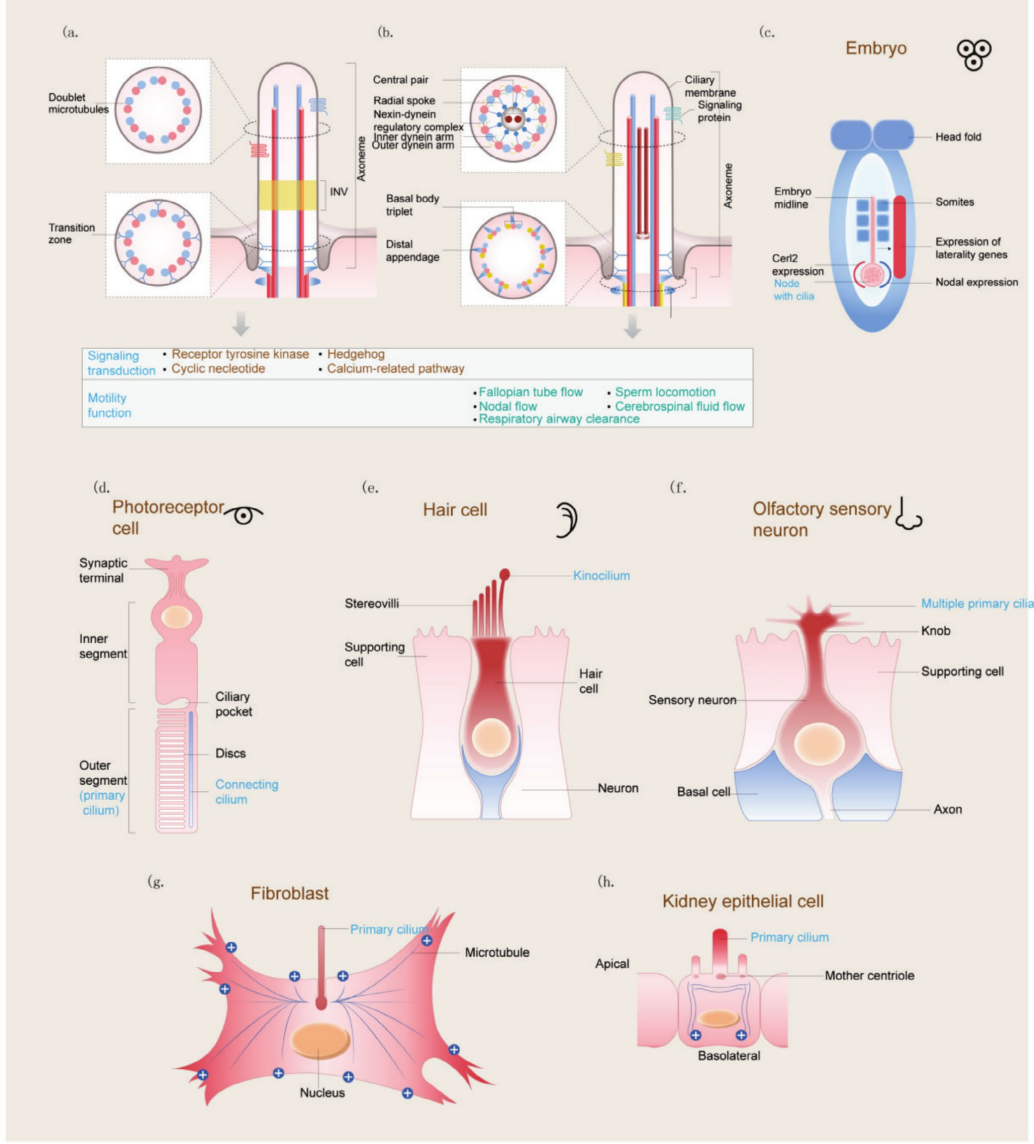
Structure of cilia and their main subcompartments. (a) All cilia originate from a basal body, which is typically composed of triplet microtubules and features subdistal and distal appendages. (b) In motile cilia, axonemes often include additional structures and proteins, such as the central pair and axonemal dyneins. Nodal cilia are motile yet lack a central pair of microtubules. Key cell signalling functions and roles in motility are highlighted. (c–f) Diagrams of various vertebrate cell types showcasing the different types of non‐motile, sensory cilia present in these cells. (g, h) Illustrations of a fibroblast and a polarised kidney epithelial cell, respectively, indicating the presence and absence of a ciliary pocket, as well as the distinct cytoplasmic microtubule organisation.

## 1 Function of Primary Cilia in Acquired Heart Disease

Primary cilia have become the main research focus because of their distinct features from motile cilia and wide distribution in mammalian tissues. The dysfunction of cilia in the cardiovascular system has previously focused on the occurrence of congenital heart diseases, during which cilia‐related genes are frequently mutated. However, primary cilia are now recognized as critical regulators of the pathogenesis of acquired heart disease (heart failure, cardiac fibrosis, arrhythmia, etc.), and the aetiology of this association remains a research focus [2]. We discussed the architecture and function of primary cilia and elaborate how impaired cilia‐associated structures and processes impact signalling, ciliogenesis, compartmentalisation (or gating) and dynamic trafficking, all of which contribute to acquired heart diseases [3]. For instance, our previous study indicated the depletion of mixed‐lineage leukaemia protein 2 (MLL2), a histone methyltransferase, leads to ciliary abnormalities, accompanied with downregulated actin‐associated proteins, altered actin dynamics, and increased vesicle transportation to the basal body, providing novel insights into the role of MLL2 in the process of cardiogenesis [4].

The function of primary cilia has been discussed in various tissues and is closely related to many physiological and pathological processes. Specifically, malfunction or structural changes in primary cilia are aetiologies of ciliopathies, which are associated with myocardial phenotypic abnormalities. For example, several ciliopathy syndromes are accompanied by heart abnormalities. As revealed by a genetic screen in fetal mice, primary cilia and the signals they transmit play crucial roles in the development of congenital heart diseases [5].

### 1.1 Function of Primary Cilia in Myocardial Fibrogenesis

**FIGURE 2:**
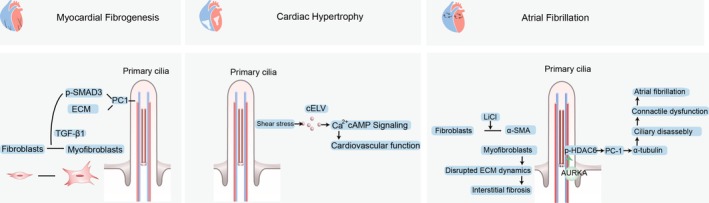
Roles of primary cilia and the signals they transmit in the development of myocardial fibrogenesis, cardiac hypertrophy, and atrial fibrillation. Left, Fibroblasts can differentiate into myofibroblasts in response to TGF‐β1. TGF‐β1 stimulation via both paracrine action in the heart and exogenous action on primary cultured fibroblasts activated the phosphorylation of SMAD3 and the transcription of the fibronectin and collagen type I and III genes. Middle, Vesicles derived from cilia are secreted at an accelerated rate under fluid shear stress. Blockage of ciliary protein, which is required for cELV generation with shRNA, led to blunted cELV secretion and left ventricular hypertrophy. Right, under pathological conditions such as atrial fibrillation (AF), fibroblasts exhibit increased proliferation and differentiation into α‐smooth muscle Actin (αSMA)‐expressing myofibroblasts. This disrupts ECM dynamics, ultimately leading to interstitial fibrosis within the atria. AF patients presented increased HDAC6 activity and reduced levels of acetylated α‐tubulin in left atrial tissues. HDAC6 activity is activated by the interaction of aurora kinase A (AURKA), and neural precursor cells express developmentally downregulated protein 9 (NEDD9) via phosphorylation. LiCl prompts the reversion of αSMA‐positive myofibroblasts into αSMA‐negative fibroblasts.

After tissue damage, such as ventricular apical resection, ischemia/reperfusion, damaged areas are replenished with cardiac fibroblasts with a primary cilium [6]. Similarly, in damaged human hearts, accumulated ciliated fibroblasts also appear adjacent to injured areas in human hearts with ischemic cardiomyopathy. As one of the most enriched cell types in the heart, fibroblasts function fundamentally in extracellular matrix (ECM) homeostasis and wound healing. Upon disease‐associated stress, fibroblasts can differentiate into myofibroblasts in response to TGF‐β1, which are specifically involved in ECM protein secretion and facilitate fibrogenesis. This process is characterised by the production, secretion and clearance of ECM components, including fibronectin, collagen types I and III, and increased matrix metalloproteinase activity. TGF‐β1 stimulation via both paracrine action in the heart and exogenous action on primary cultured fibroblasts activated the phosphorylation of SMAD3 and the transcription of the fibronectin and collagen type I and III genes. Inhibition of SMAD3 signalling abrogates the fibrotic process elicited by TGF‐β1 in cultured fibroblasts [7] (Figure 2). Moreover, primary cilia in fibroblasts exert fundamental effects on the TGF‐β1 response, SMAD3 activation, ECM secretion and fibrosis [8]. For example, inactivation of primary cilia with siRNAs targeting PC1 in fibroblasts is incapable of promoting the production of collagen when stimulated with TGF‐β1 [9]. Similarly, knockdown of PC1 or IFT88 failed to transform fibroblasts into myofibroblasts, which is a prerequisite for cardiac remodelling. Instead, these mice displayed exacerbated cardiac hypertrophy, as well as altered scar structure. In addition to native cardiac fibroblast proliferation, it is increasingly acknowledged as a significant contributor to the pool of fibroblasts involved in perivascular and subendocardial fibrosis. Knocking down IFT88 in endothelial cells led to the absence of primary cilia, which are unable to induce EndMT in vivo. However, these cells may be more prone to EndMT when exposed to lower levels of stress than typically required [10].

### 1.2 Function of Primary Cilia in Cardiac Hypertrophy

Primary cilia play critical roles in cardiac development and diseases by sensing mechanical signals (e.g., blood flow shear stress), regulating developmental pathways (e.g., Hedgehog signalling), and maintaining cardiomyocyte polarity, with dysfunction contributing to congenital heart defects (e.g., hypoplastic left heart syndrome) and cardiomyopathies. Cardiac hypertrophy is an adaptive alteration in response to dramatic stresses that is either autonomous or noncell autonomous. This process is essential for survival. However, long‐term stress and consequent excess cardiac hypertrophy and cardiac remodelling are detrimental and ultimately lead to heart failure and sudden cardiac death [11]. Cardiomyocytes can sense mechanical signals, such as hemodynamic stress, to transmit stress signals into cellular growth signals and induce hypertrophy. However, the molecules involved in mechanical sensing remain unknown. Primary cilia serve as promising mechanosensory targets, which have not been clearly documented. One plausible explanation is that they work via ciliary extracellular‐like vesicles (cELVs) (Figure 2). Vesicles derived from cilia are secreted at a normal rate under normal conditions, but at an accelerated rate under fluid shear stress [12]. Blockage of ciliary protein, which is required for cELV generation with shRNA, led to blunted cELV secretion and left ventricular hypertrophy, accompanied by a decreased left ventricular ejection fraction, low blood pressure, and ultimately cardiovascular collapse [3].

### 1.3 Function of Primary Cilia in Atrial Fibrillation

Atrial fibrillation (AF) patients presented increased HDAC6 activity and reduced levels of acetylated α‐tubulin in left atrial tissues [13]. HDAC6 is activated by the interaction of aurora kinase A (AURKA), and neural precursor cells express developmentally downregulated protein 9 (NEDD9) via phosphorylation. In addition, subsequently, HDAC6 deacetylates acetylated α‐tubulin, resulting in the quick disassembly of the ciliary axoneme. As revealed by a recent transcriptome study, genes related to cilia assembly (e.g., IFT proteins) were significantly downregulated, and genes related to cilia collapse (e.g., NEDD9 and LIMK2) were upregulated in the left atrial tissue of patients with AF. As a possible link between ciliary dynamics and AF, the modulation of cilia assembly might provide new avenues for the treatment of AF. LiCl is well known for its ability to extend primary cilia in several cell types but to suppress ECM genes induced by TGF‐β1 in NHCF‐A cells. Moreover, LiCl prompts the reversion of αSMA‐positive myofibroblasts into αSMA‐negative fibroblasts [13] (Figure 2). These findings indicate that the LiCl‐induced elongation of primary cilia might restore existing atrial fibrosis in AF patients if this effect is replicated in vivo. Lithium is known to increase cilia length by activating α‐tubulin acetyltransferase‐1 and inhibiting GSK‐3β; however, it has diverse effects on various cellular signalling pathways, and its precise mechanism of action remains unclear. From a clinical perspective, lithium may offer benefits to AF patients; low doses of lithium appear to have antiarrhythmic effects, and as a potent inhibitor of GSK‐3β, it can prevent myocardial apoptosis in the diabetic myocardium. However, similar to most antiarrhythmic drugs, lithium toxicity can lead to adverse electrocardiographic and proarrhythmic changes.

The AURKA/NEDD9 axis represents a promising therapeutic target in AF. Alisertib (MLN8237), an oral selective inhibitor of AURKA, is currently being studied for the treatment of malignancies and may also hold potential for treating fibrosis in AF. Targeting the NEDD9/AURKA/HDAC6 axis is an appealing novel approach for treating fibrosis in AF, as it could restrict the activation of AURKA/HDAC6 and the subsequent disassembly of primary cilia to specific cardiac cell types [14].

### 1.4 Function of Primary Cilia in Cardiac Regeneration

Mechanical stress generated by cardiac contraction and haemodynamic forces can be sensed by primary cilia on ventricular endocardial cells and transduced to the flow‐responsive transcription factor KLF2, followed by activation of Notch1 and its downstream effectors ephrin b2a (EFNB2a) and neuregulin 1 (NRG1) in the endocardium for the modulation of trabeculation initiation. Primary cilia function as a bridge between haemodynamics and Notch signalling [15]. Primary cilia on the endothelium are associated with slow fluid sensation and transduce shear stress signals to downstream functional responses. For example, in the embryonic zebrafish endocardium, primary cilia detect shear stress and promote cardiac trabeculae by activating Notch1b. Primary cilia exist not only in the endocardium, but also in the epicardium [16]. In addition, the number and length of primary cilia undergo dynamic alterations in response to changes in blood flow over the course of embryonic development and heart regeneration. The knockdown of Tnnt2a, a sarcomeric gene, markedly inhibited contractile function, impeded blood flow, and reduced the number of primary cilia [17]. Tricaine treatment can abrogate the increase in the number of primary cilia by inhibiting blood flow in ablated hearts. Since IFT proteins are involved in cilia assembly, several IFT mutants and morphants destroy primary cilia formation in the heart. Specifically, cardiac IFT88 morphant ablation decreases KLF2A and KLF2B expression, suppresses Notch signalling, and inhibits cardiomyocyte proliferation. This suggests that primary cilia play a critical role in haemodynamic regulation and cardiac regeneration [18]. Moreover, IFT88 regulates angiogenesis through microtubule‐based cellular processes, independent of its role in ciliogenesis [19].

The transient receptor potential (TRP) family, a group of calcium‐permeable ion channel proteins, especially TRPV4, functions as signal transductor. TRPV4 is widely involved in heart valve development, arterial dilation, and vascular pressure, endothelial Ca^2+^ influx, and vasodilatory responses in the cardiovascular system. During valve formation, TRPV4 can interact with primary cilia. TRPV4‐depleted hearts presented markedly inhibited KLF2A and Notch expression, impaired expression of early cardiac transcription factors, and blunted cardiomyocyte proliferation and reprogramming, ultimately resulting in reduced heart regeneration [20]. Given the important role of cilia in the context of cardiac damage in mammals, cilia response modulation serves as a promising target for restoring cardiac regeneration.

## 2 Outlook

Future studies are expected to explore the integrative role of cilia‐mediated signalling in the cardiovascular system in greater depth. While signalling pathways, such as the Hedgehog, Wnt, and Notch pathways, have been individually implicated in cardiac development and remodelling, their concerted interactions within the ciliary microdomain remain insufficiently characterized. Deciphering how primary cilia orchestrate signal convergence and divergence under physiological and pathological conditions is critical. With the advent of spatially resolved omics technologies and advanced in vivo imaging, constructing a comprehensive network model of ciliary signalling and its spatiotemporal regulation across diverse cardiac cell types will become increasingly feasible.

Building on this molecular understanding, the therapeutic potential of targeting cilia is an emerging frontier. Pharmacological modulation of cilia structure and function—such as increasing ciliary stability to suppress maladaptive fibroblast activation—has shown promise in preclinical studies. Compounds, such as lithium and HDAC6 inhibitors, may indirectly influence fibrotic or inflammatory processes by restoring ciliary integrity. Future efforts should focus on the development of cilia‐specific therapeutics with high tissue selectivity and minimal systemic toxicity. These strategies, combined with gene editing or RNA‐based interventions, may open new avenues for the precise modulation of cilia in acquired heart diseases.

Another promising direction lies in the intersection between cilia and cardiac regeneration. Primary cilia play a pivotal role in sensing biomechanical cues and transducing them into regenerative signals via KLF2, Notch, and other pathways. Observations in model organisms suggest that cilium‐dependent signalling can promote cardiomyocyte proliferation and endocardial remodelling. Extending these findings to adult mammalian hearts remains a key challenge. Understanding how the mechanical microenvironment, flow dynamics, and ciliary machinery converge to regulate regenerative capacity could inform the design of bioengineered therapies or mechanical stimulation protocols to enhance endogenous repair mechanisms after cardiac injury.

Finally, translating these insights into clinical applications holds considerable promise for precision cardiovascular medicine. Alterations in ciliary dynamics, regardless of length, protein composition, or signalling capacity, may serve as early biomarkers of disease susceptibility or therapeutic response. Integrating cilia‐related parameters into patient stratification models could refine diagnostics and guide individualised treatment plans for conditions such as atrial fibrillation, myocardial fibrosis, or heart failure. Ultimately, realising the clinical utility of cilium‐targeted strategies will require multidisciplinary collaboration to bridge fundamental biology with translational cardiology.

## Author Contributions

**Yang Yang**, **Kaidi Ren**, and **Yi Luan:** conceptualised and wrote the manuscript and created the figures. **Xingjuan Shi**, **Yang Yang**, and **Kaidi Ren:** contributed to the writing of the manuscript. **Yi Luan**, **Xingjuan Shi**, and **Yang Yang:** reviewed and modified the manuscript. All authors approved the final version of the manuscript.

## Conflicts of Interest

The authors declare no conflicts of interest.

## Data Availability

The data that support the findings of this study are available on request from the corresponding author. The data are not publicly available due to privacy or ethical restrictions.
